# River Food Web Response to Large-Scale Riparian Zone Manipulations

**DOI:** 10.1371/journal.pone.0051839

**Published:** 2012-12-20

**Authors:** J. Timothy Wootton

**Affiliations:** Department of Ecology and Evolution, The University of Chicago, Chicago, Illinois, United States of America; Institute of Marine Research, Norway

## Abstract

Conservation programs often focus on select species, leading to management plans based on the autecology of the focal species, but multiple ecosystem components can be affected both by the environmental factors impacting, and the management targeting, focal species. These broader effects can have indirect impacts on target species through the web of interactions within ecosystems. For example, human activity can strongly alter riparian vegetation, potentially impacting both economically-important salmonids and their associated river food web. In an Olympic Peninsula river, Washington state, USA, replicated large-scale riparian vegetation manipulations implemented with the long-term (>40 yr) goal of improving salmon habitat did not affect water temperature, nutrient limitation or habitat characteristics, but reduced canopy cover, causing reduced energy input via leaf litter, increased incident solar radiation (UV and PAR) and increased algal production compared to controls. In response, benthic algae, most insect taxa, and juvenile salmonids increased in manipulated areas. Stable isotope analysis revealed a predominant contribution of algal-derived energy to salmonid diets in manipulated reaches. The experiment demonstrates that riparian management targeting salmonids strongly affects river food webs via changes in the energy base, illustrates how species-based management strategies can have unanticipated indirect effects on the target species via the associated food web, and supports ecosystem-based management approaches for restoring depleted salmonid stocks.

## Introduction

Conservation concerns often focus on select species when assessing causes of population decline because particular species frequently function uniquely as resources for humans, as dominant players in ecosystems (e.g. keystone or foundation species), or as aesthetically attractive components of nature (e.g. “charismatic megafauna”). Hence, management plans implemented to address conservation goals tend to be based on the autecology of target species both as a natural result of the initial focus of concern, and because a focal species approach seemingly simplifies the problem [1]–[4]. Effects of environmental factors impacting focal populations and of management activities aimed at focal species usually have some impact on other components and properties of the associated ecosystem [3]–[9]. Such impacts can be transmitted indirectly to target species because of the web of interactions among species and ecosystem components [2], [10]–[13], hence taking a broader view of environmental impacts and potential management strategies may be important in understanding and successfully predicting effects of management activities.

Understanding human impacts on rivers is essential because of their multi-faceted role both in affecting human populations and in maintaining resident biodiversity. As the interface between terrestrial and river systems, riparian zones can play a particularly important role in mediating human impacts on rivers [14]. For example, in coastal areas, the health of economically important wild salmonid stocks may be associated with the condition of riparian habitat [1]. The hypothesized mechanisms underlying such associations, such as changes in river geomorphology arising from fallen trees [15]–[17], have often been viewed from a focal species perspective, and have shaped recent management schemes to restore depleted salmonid stocks. For instance, altering riparian habitats to develop a conifer-dominated shoreline is being explored as an approach to introduce more decay-resistant wood into rivers, thereby providing better hydrological conditions for salmon [1]. Little information exists on the outcomes of this riparian conversion approach [18].

Altering riparian habitat can affect other components of river ecosystems through a variety of mechanisms, however, including biologically important changes in the intensity and spectrum of solar radiation (ultraviolet (UV), infared, and photosynthetically active (PAR)), alterations of nutrient regimes, effects on the energy base, and modifications of both terrestrial and aquatic habitat structure [5], [10]–[13], [19]–[28]. Such impacts could indirectly affect salmonid populations via changes in the food web structure impacting juvenile fish rearing in the river [22], [29]. Elucidating the role that impacts on other food web components play in affecting target salmonid populations can help to anticipate unexpected consequences of single-species targeted management, and may suggest alternative management approaches [8], [22], [27], [30]–[32]. Experiments probing such mechanisms are usually logistically limited to small scales in order to achieve adequate replication for statistical analysis, but such experiments may not translate to management-relevant scales. Using an unusual replicated large-scale manipulation of riparian habitat, I show that riparian management can have indirect effects on salmonid populations via impacts on the associated river food web. Hence, an ecosystem-based perspective is necessary to anticipate the effects of riparian management approaches, and can reveal alternative strategies for salmond conservation.

## Results

**Figure 1:**
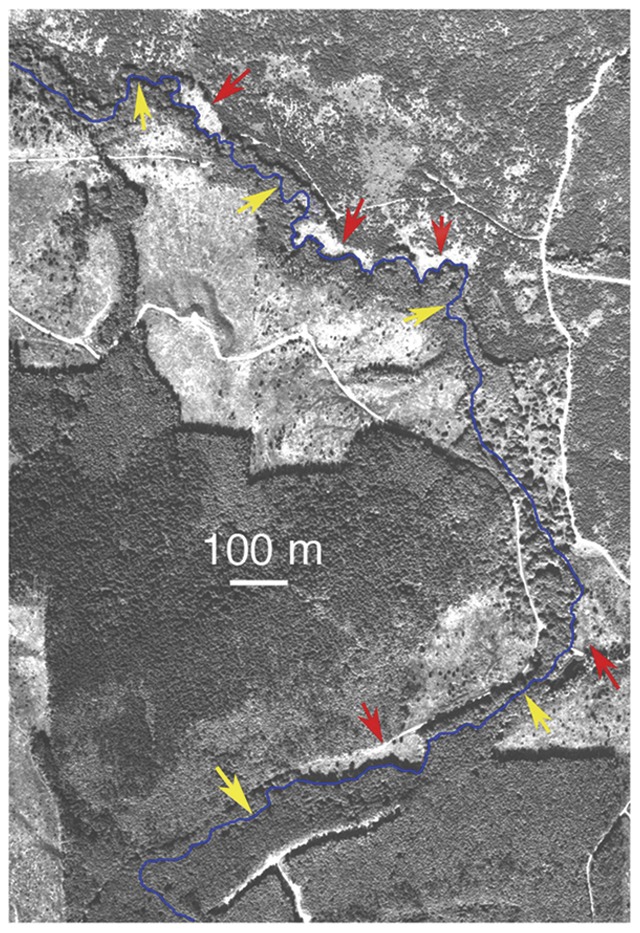
Aerial photograph of riparian manipulations along the South Fork Pysht River, Olympic Peninsula, Washington State, USA. Red arrows indicate manipulated reaches, yellow arrows indicate intervening unmanipulated (control) reaches sampled.

In an effort to enhance salmon stocks by increasing long-term delivery of conifer wood to rivers, a series of five 100–300 m long riparian manipulations were implemented along the South Fork Pysht River, located on the Olympic Peninsula, Washington state, USA, in 1994 (Fig. 1). In these experimental reaches, the riparian vegetation from one bank of the river, which was initially dominated by nitrogen-fixing red alder (*Alnus rubra*), was removed and replaced with conifer seedlings. The riparian zone of intervening reaches of river was not manipulated, and these reaches served as controls that were paired with the closest manipulated reach for statistical analyses. As would be expected from a tree removal, riparian manipulations caused a 40% decline in canopy cover.

Reducing riparian canopy cover caused trapped leaf litter to decline by two-thirds, UV radiation to increase 12-fold, and PAR to increase 42-fold (Table 1). Other physical variables did not differ statistically with treatment, including water temperature, benthic substrate, and variation in water depth (Table 1). Nutrients (NO_3_, NO_2_, NH_4_, PO_4_), dissolved organic carbon (DOC), and silicates, which are associated with elevated sediment input [33], did not vary significantly with riparian treatment (Table 1). No upstream-downstream gradients in measured variables were detected, except for variation in water depth, suggesting that aggregate effects of the manipulation were not manifesting themselves at larger scales.

**Table 1:** Summary of physical and chemical variables measured in experimental and control reaches.

Variable	Manipulated	s.d.	Control	s.d.	P*
**Temperature (°C)**	13.4	0.45	13.3	0.42	0.48
**Canopy Cover (%)**	53.33	2.88	89.06	1.80	***<.0001***
**PAR (µmol m^−2^ sec^−1^)**	268.8	545.2	6.47	4.99	***0.03***
**UV (µmol m^−2^ sec^−1^)**	19.58	12.49	1.70	1.41	***0.02***
**Leaf Input (g m^−1^ hr^−1^)**	4.92	2.55	14.12	5.70	***0.04***
**% Riffle**	29	6.21	23.1	11.05	0.47
**% Pool**	35.6	10.16	46.5	14.44	0.29
**Variance in Water Depth (cm)**	216.82	93.13	302.6	124.54	0.26
**% Coarse Woody Debris**	6.2	4.43	1.8	1.3	0.06
**% Cobble**	62	10.03	53.6	8.44	0.14
**% Silt/Sand**	2.8	2.17	2.7	2.01	0.93
**SiOH_4_ (µM)**	123.05	13.43	115.45	28.68	0.53
**DOC (µM)**	5.09	2.93	5.23	4.24	0.96
**PO_4_ (µM)**	0.171	0.012	0.172	0.015	0.83
**NO_3_ (µM)**	7.41	1.15	7.51	1.62	0.85
**NO_2_ (µM)**	0.030	0.005	0.028	0.006	0.36
**NH_4_ (µM)**	0.71	0.23	0.58	0.26	0.51
**N∶P**	49.15	6.01	48.96	13.75	0.96

* Statistical comparisons based on paired t-tests.

N = 5 per treatment.

**Figure 2:**
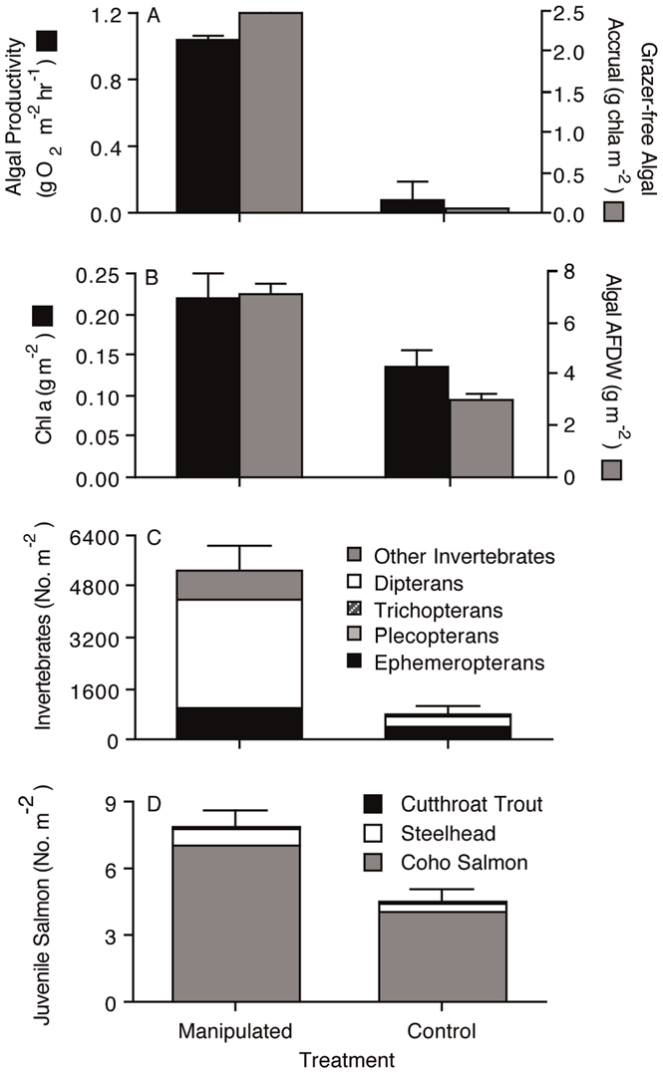
Biological responses to riparian manipulations (mean ± 1 se). (A) Algal productivity, based on light-dark oxygen production (black bars) and grazer-free algal chlorophyll *a* accrual (gray bars). (B) Algal standing crop estimated from either ash-free dry mass (gray bars) or chlorophyll *a* (black bars). (C) Aquatic invertebrate densities. (D) Juvenile salmonid densities, distinguished by species.

Biological components responded strongly to the treatment. Algal production increased 13-fold (P<0.001), grazer-free algal accrual increased 55-fold (P<0.001), algal standing biomass increased by 60% (P<0.001), and algal standing chlorophyll *a* increased 2.4-fold (P<0.02) in manipulated reaches relative to controls (Fig. 2a, b). The difference in algal biomass on invertebrate exclusion tiles compared to tiles accessible to invertebrates (Fig. 2a, b) suggests strong grazer impacts on algal standing crop, particularly in manipulated areas where algae increased by more than an order of magnitude.

Aquatic insect abundance increased 7-fold (P<0.002), with all major taxonomic groups showing elevated populations (Fig. 2c). Densities of juvenile salmonids, primarily coho salmon (*Oncorhynchus kisutch*; 90.7%) with some steelhead (*O. mykiss*) and cutthroat trout (*O. clarkii*), increased on average 77% in manipulated reaches (Fig. 2d, P<0.03), and exhibited an increasing trend through time (r = 0.799, P<0.02). Trout comprised a larger fraction of the fish community in downstream than in upstream sites (r = 0.781, P<0.01). Salmonid densities correlated strongly with algal production (r = 0.738, P<0.015), but not with any of the physical or habitat variables measured (all P>0.05).

**Figure 3:**
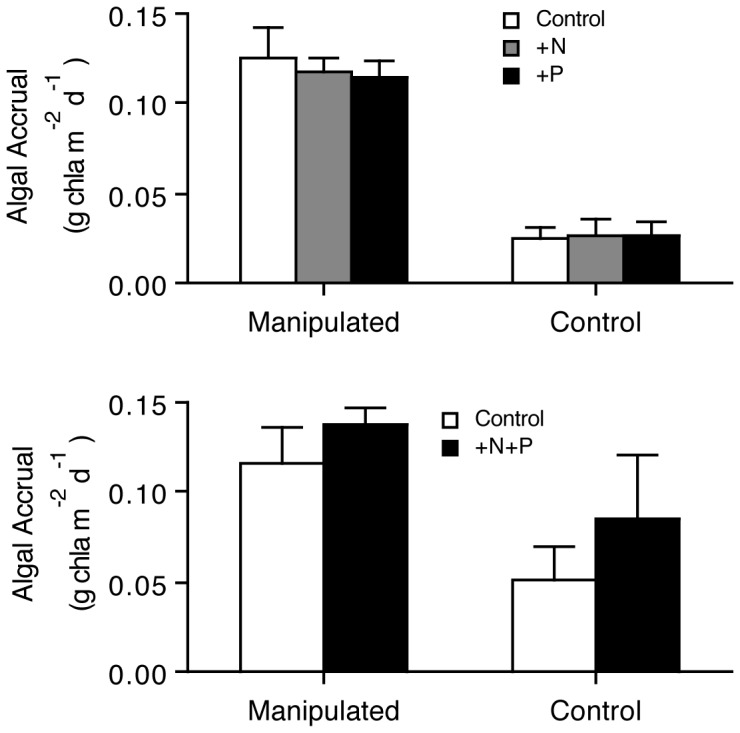
Algal Responses (mean ± 1 se) to Nutrient Addition in River Reaches with Intact (Control) and Manipulated Riparian Vegetation. All responses measured as rates of chlorophyll *a* accrual. Top: results from single nutrient manipulations of nitrogen (as NaNO_3_) and phosphorous (as NaH_2_PO_4_). Bottom: results from simultaneous nutrient additions using Osmocote pellets (18 N∶ 6 P).

There was no evidence of nutrient limitation on algal production in experiments manipulating nutrients (P>0.5 for N, P), but algal accumulation was 5 times higher in treated than control reaches (Fig. 3a, P<0.001). In experiments manipulating N and P together, there was also no statistically significant effect of nutrients (Fig. 3b, P>0.05), although chlorophyll *a* levels in the presence of nutrients tended to be higher in both riparian treatments, a pattern suggestive of weak nutrient co-limitation [34].

**Figure 4:**
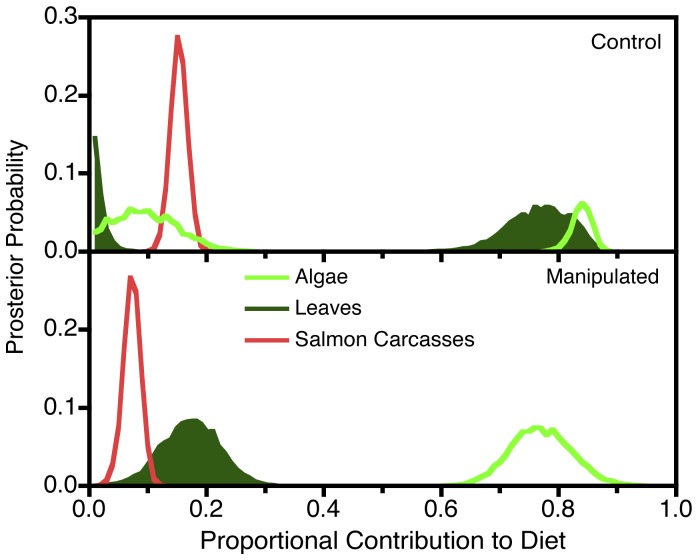
Estimated Contributions of Different Energy Sources Entering the River to Juvenile Salmonids Based on Stable Isotope Analysis. Graphs show Bayesian posterior probabilities of different fractional diet compositions, derived from the MixSIR algorithm [65], assuming a two trophic level energy transfer to salmon via aquatic invertebrates. Top: control reaches. Bottom: treatment with reduced riparian vegetation.

Stable isotope analysis reinforced the hypothesis that salmonid responses to the manipulation arose from changes in the energy base. Isotopic composition shifted significantly (P<0.05) among treatments for both algae and juvenile salmon (Table 2). The shift in algal isotopes is expected because increased carbon fixation via algal photosynthesis in manipulated areas depletes the thermodynamically favored (lighter) isotope of carbon [35], [36]. In the reduced riparian zone treatment, algal-derived production provided the dominant (70–80%) energy base supporting juvenile salmonids (Fig. 4), whereas leaves (15–20%) and salmon carcasses (10%) provided smaller contributions. The preponderance of evidence from stable isotopes indicated that the manipulation shifted the energy base supporting salmon from allochthanous terrestrial production (leaves) to autochthanous algal production (Fig. 4). This scenario was three times more likely than a scenario in which juvenile salmon depended on algal production in both treatments. In either case, the dominance of the algal contribution in the manipulation supports the hypothesis of shifts in salmon populations via food-web interactions.

**Table 2:** Stable isotope ratios of nitrogen (^15^N/^14^N) and carbon (^13^C/^12^C) in tissues of potential food web energy sources, and of juvenile salmonids from different riparian zone treatments.

	δN		δC	
Source (N)	Mean	s.d.	Mean	s.d.
**Adult Salmon Carcasses (6)**	14.04	0.54	−17.6	0.75
**Leaves (4)**	−0.68	0.34	−29.20	1.74
**Algae: Control (5)**	−0.03	0.38	−28.15	2.51
**Algae: Manipulated (5)**	0.45	0.24	−24.61	0.58
**Juvenle Salmon: Control (10)**	7.40	1.33	−24.1	1.03
**Juvenile Salmon: Manipulated (10)**	7.40	1.56	−23.4	1.01

δ values represent differences in isotopic ratios from international standards (atmospheric N, Vienna Pee Dee Belemnite C).

## Discussion

The intended response to riparian conversion management, enhanced conifer dominance and increased input of recalcitrant conifer logs into the river, is expected to arise only after extended (half century) time scales. Therefore, exploring short-term responses to riparian treatments reveals alternative indirect mechanisms of riparian management that affect targeted salmonid species. The pattern of results indicates that manipulations of riparian vegetation affected juvenile salmonids via food web interactions. The primary differences detected were in the energy base of the food web (light-driven algal production versus leaf input). This change corresponded to increases in algal production. The abundance of food web components increased in association with increases in algal production, and isotopic analysis revealed that algal-derived energy largely contributed to juvenile salmon body tissue where riparian manipulations were imposed. Other postulated negative effects of riparian manipulation, including increases in damaging UV radiation [5], [25], and reductions in leaf energy input [26], either were overwhelmed by the effects of increasing algal production or did not arise because of the ameliorating effects of the control reaches or of the environmental setting.

The lack of change in water temperature in response to the treatment is somewhat surprising given prior studies [12], although it has been observed in other high light situations [37]. Three factors may contribute to this result. First, the relatively cool and cloudy conditions typical of the Olympic Peninsula may have contributed to reduced severity of temperature effects, although most temperature measurements were made during late summer when days tended to be sunny and air temperatures are generally 5–10°C above the water temperatures. Second, the interspersion of shaded control reaches may have helped to offset temperature increases. Because air temperatures were warmer than water temperatures even in shaded reaches, however, the intervening reaches probably do not serve to cool water temperatures. Finally, the study reaches have a porous alluvial bottom of gravel, cobbles, sand and mud, with little exposed bedrock. This situation permits substantial subsurface flow through the bottom gravel, which may enforce a fairly constant water temperature because of the high thermal inertia of the riverbed. Observations of other reaches in the river characterized by bedrock and high solar radiation show detectible temperature increases (J. T. Wootton, unpublished data) [37]. Hence, the results of riparian manipulations may vary with characteristics of the stream bottom.

The riparian manipulations might have been expected to alter nutrient-algal relationships [30]–[32], [38], but these did not arise. Although the removal of nitrogen-fixing alders along the river could affect nutrient levels, the scale of the removals is relatively small compared to the entire watershed that affects nutrients processes. The lack of nutrient response probably arises in part from the cool cloudy conditions of the area, coupled with riparian shading, both of which could lead to light limitation, but periphyton might be expected to respond strongly to nutrient addition in manipulated treatments where light levels were increased. Like most situations where riparian conversion is likely to be implemented, the study river flows through a landscape subject to timber harvest. As in other landscapes that have experienced timber harvest [19], nutrient levels in this river are elevated relative to nearby rivers with intact watersheds flowing through Olympic National Park [33] because of some combination of reduced nutrient retention in the watershed and because harvested watersheds are dominated by nitrogen-fixing alders [26]. Hence, the higher nutrient levels in the river may have precluded nutrient limitation even in reaches with high light availability. These results contrast with results of management targeting increased food web production through nutrient additions in British Columbia lakes and streams [30]–[32], which were designed to counteract reduced nutrient input by migrating spawning salmon following stock depletion. My results suggest that this strategy may depend on the watershed context, with nitrogen fixation in alder-dominated watersheds muting the benefits of fertilizer addition.

Although not statistically significant, large woody debris tended to be more common in treatment reaches. The direction of this trend might seem surprising given the lower tree stock available in treatment reaches, but direct observations suggest it could arise if tree removal from the riparian zone reduced protection from wind, increasing the chance of blowdowns [39], [40]. If so, then this mechanism represents another unintended effect of riparian conversion management. Given that salmon production has been associated with the presence of large woody debris [15], [39], [41], a possible alternative hypothesis for the differences between treatments is that salmon responded to higher levels of large woody debris rather than increases in benthic production. Available evidence does not support this hypothesis. First, although large woody debris tended to be more prevalent in manipulated reaches, this did not translate into the geomorphic impacts presumed to affect salmonids, as changes in characteristics such as water depth profile, bottom substrate, or pool-riffle composition did not arise (Table 1). Second, salmon abundance did not correlate significantly with the relatively slight variation in large woody debris, in contrast to its strong association with measured algal productivity of a reach.

Biotic and abiotic components of the ecosystem probably moved between manipulated and unmanipulated reaches in this experiment to some extent. Although this situation is most obvious in river settings, movements among treated and untreated areas of at least some ecosystem components occur in all field experiments. Despite the potentially high mobility of fish, shallow riffles appeared to discourage their movement among pools during the summer growing period, and fish generally moved deeper into pools rather than through riffles when startled. These observations and interpretation are supported by movement studies in this (J. T. Wootton, unpublished data) and other areas [42], and by the distinct differences in stable isotope composition in the tissues of salmon from the different reaches (Table 2). In general, movements among treatments make field experimental results conservative, relative to actual effect sizes, but limitations in the ability of field experiments to precisely identify “the” effect size does not compromise the proven power of field experiments to qualitatively reveal key linkages among ecosystem components. In situations with strong directional biases in movement, such as might occur in rivers as a result of water flow, it is possible that treatment effects might accumulate and affect experimental results. This hypothesis can be tested readily by exploring whether treatment differences tend to increase with distance. There is little evidence for such effects in this study, because of the 34 variables considered, in only 1 case (rate of leaf trapping) did the difference between paired manipulated and control reaches change significantly with downstream position. This single result may have arisen by chance, as overall leaf trapping did not vary significantly with downstream position and there is no obvious mechanism by which removal of riparian vegetation would generate a higher difference in leaf trapping rate between treatments in downstream compared to upstream reaches.

The results of this study focus on ecosystem conditions relevant to the stream-rearing juvenile stage of salmonids, as do many of the concerns about human impacts and the management strategies targeting the stream phase of the life cycle. Therefore, the responses of such a manipulation may be different for other salmon species that spend little time in rivers. Studies of the stream-phase in the salmon life cycle implicitly assume that increased juvenile performance results in higher abundance of adult spawners [2]. Other life cycle stages are likely to be sensitive to different ecological factors, however, and may also play critical roles in determining salmon population dynamics, perhaps muting or even counteracting patterns arising during the juvenile stage [11], [43]–[45]. Hence, connecting the response of juvenile fish to adult return rates is an important goal for future research [2].

This study illustrates that taking a broader ecosystem-based perspective may increase the effectiveness of developing management strategies to enhance depleted species or control invasive species. The results reported here demonstrate that management targeting a single species has system-wide impacts, and that these impacts indirectly affect the target species through entirely different mechanisms than those underlying the management plan. Specifically, altering riparian zone vegetation caused shifts in the energy base of the river food web, which ultimately affected juvenile salmonid abundance. Over longer time scales (40–70 years) the intended direct effects of increased falling wood on structural characteristics of the river are likely to occur [41], [46], [47]. As the riparian canopy closes in, further food web impacts may also become apparent, however, as conifer seedlings mature along manipulated reaches, causing differences in leaf litter quality (conifer needles versus deciduous leaves [48]) and in seasonal light intensity in treated compared to untreated reaches. Deciduous leaves often provide better food resources for aquatic invertebrates [48], and seasonal leaf fall may permit higher algal production at some times of the year in areas with deciduous vegetation [49], [50] hence changing the riparian forest composition could further change food web processes relevant to rearing salmonids. The food web consequences of these differences need to be more thoroughly explored. Additionally, by taking a food web based perspective, alternative management approaches may be revealed [8], [27], such as the enhanced ecosystem productivity approach in natural streams currently being implemented in British Columbia [30]–[32]. Beyond such nutrient additions, the results suggest that, in geomorphologically appropriate areas, introducing limited productivity hotspots in the context of intact riparian corridors may be a useful approach to restoring depleted stocks of salmonids whose juveniles rear in rivers. Hence, in situations where tradeoffs exist in the amount of riparian corridor protection possible, conservation regulations may need to be shifted somewhat to focus more on tributaries lacking fish, where undesirable sediment input is higher and productivity benefits are lower.

## Study Site and Methods

### Ethics Statement

All necessary permits were obtained for the described field studies. The study sites are owned by Merrill and Ring L. P. (Pysht Tree Farm), which provided permits allowing research on their land. With the exception of salmonids, which were surveyed via visual observation, the research involved no endangered and protected species. The research plan was reviewed and approved by the Washington State Department of Fish and Wildlife, which issued permits to collect salmon for stable isotope analysis and otherwise determined that it did not require any other permit.

### Study Site

A set of five 100–300 m long riparian manipulations were established along the South Fork Pysht River, located on the Olympic Peninsula, Washington state, USA, in 1994 (Fig. 1). In experimental reaches, the red alder-dominated riparian vegetation from one bank of the river was removed and replaced with conifer seedlings. Alder removal extended back at least 50 m from the riverbank. To minimize undesirable effects of sediment input, the manipulation was carried out in a low gradient stretch of river with shallowly sloping banks, and the roots of the removed trees were left in place. The riparian zone of intervening reaches of river was not manipulated, and these reaches served as controls that were paired with the closest manipulated reach for statistical analyses. In the experimental areas, the river is relatively small, typically ∼5 m wide and ∼50 cm deep during summer base flow, and riffles between intervening pools are quite shallow (∼5 cm). The river is presently not stocked with salmon or trout, but was stocked with chum salmon (*Oncorhynchus keta*) several decades ago.

Between 1997–2004, data were collected during summer base flow from each experimental and control reach on a variety of physical variables, water chemistry, primary productivity, rate of leaf trapping, nutrient limitation, and the abundance of periphyton, aquatic invertebrates, and juvenile salmonids. In each experimental and control reach, sampling was carried out in run habitats large enough to accommodate both sampling devices and small-scale experiments. Runs, stretches of non-depositional habitat characterized by laminar flow (median velocity ∼30 cm/sec) in early summer and cobble (3–10 cm diameter) bottoms were chosen because they are sites of high benthic productivity [51], [52] and are used extensively as feeding areas by juvenile salmonids at this and other sites (J. T. Wootton personal observation). During late summer base flow, the current speeds in these areas generally dropped to <10 cm/sec but the bottom remained cobble.

### Physical and Chemical Variables

At each site, canopy cover was measured as a percentage of the sky covered by leaves using a spherical densiometer, temperature was measured with a submerged thermometer at each visit during the spring-summer sampling season, photosynthetically active radiation (PAR) was measured using a LICOR LI-190SA quantum sensor, and ultraviolet radiation (250–400 nm) was measured using a Spectrum Technologies UVM UV meter. Conclusions for temperature and light levels were checked in August 2005 with submerged Onset Computer HOBO Pendant light intensity and temperature loggers placed at each site for 6 days with a logging interval of 10 min. Leaf trapping in the river was estimated by placing 15×122 cm pieces of 0.5 cm plastic mesh across the river just upstream of riffle habitats and collecting leaves trapped on the mesh after 1 hour. Leaves were sorted by taxon, dried at 70°C and weighed. This sampling method mimics natural leaf-trapping in the river, which occurs where rocks emerge at the entry of riffles, and was chosen over the more common method of capturing leaves as they fall into the stream because I was most interested in the leaf biomass retained within a given reach, which would be most available to the local stream biota. Leaf traps were placed in the river in early September at the onset of leaf fall. Habitat was measured by establishing a 100 m transect centered at the sampling site, and by recording the bottom substrate (silt, sand, mean cobble size, wood), water depth and presence of large woody debris (>5 cm diameter) below bank-full depth at 1 m intervals. I took water samples for chemical analysis by collecting water at each site using a 60 cc syringe, filtering the sample through a Whatman GF/F glass fiber syringe filter into an acid-washed bottle, and freezing the sample upon return from the field. Water samples were express-shipped to the University of Washington Marine Chemistry Laboratory, where they were analyzed for DOC, NO_3_, NO_2_, NH_4_, SiOH_4_, and PO_4_ following the methods outlined in [53].

### Primary Productivity

I assessed algal production in two ways. First, I estimated rates of primary production using light-dark chamber methods [22]. Ceramic floor tiles (7.5×7.5 cm) were incubated in the river for 1 month to accumulate a natural algal crop, then placed into a phytosynthesis chamber constructed from a 1000 ml clear polymethylpentene jar (Nalgene) with a 2.5 cm hole drilled in the top and plugged with a rubber stopper. Productivity estimates were made at ambient light on a clear day, and pairs of experimental and control reaches were always measured concurrently to control for possible temporal differences in irradiance. Initially, I covered the chambers with aluminum foil to eliminate light and estimate respiration rates. Oxygen concentrations at the beginning and the end of the respiration period were made using a YSI-55 portable oxygen probe placed through the hole in the roof of the chamber. Then I removed the foil and incubated the chamber for a half hour period, after which another oxygen measurement was taken. I estimated production as the difference in rate of oxygen change between the uncovered and covered periods. Algae from the tiles were collected for analysis of standing crop. As a second estimate of production, I also measured algal accrual in the absence of grazers. To exclude grazers, the sides were cut out of square buckets and the holes were covered with 0.05 mm^2^ mesh attached to the bucket with hot melt glue [54]. Netting of this mesh size excludes all aquatic grazers but the smallest size class of chironomids. A 15×15 cm ceramic floor tile was placed in the bucket on top of a layer of flat cobbles to keep it out of the boundary layer, and the bucket was placed in the river. The bucket sides were cleaned periodically by hand to minimize flow alteration. After 2 months, the amount of algae that accumulated on the bucket tile was estimated by scraping a 7.5×7.5 cm area, diluting the sample in 100 ml water, homogenizing the sample with a hand blender, and extracting chlorophyll *a* from a 1 ml subsample mixed with 9 ml ethanol in a black vial which was stored at −10°C for 24 hours. The sample was filtered through a Whatman GF/C syringe filter and chlorophyll *a* was measured using a Turner 450 fluorometer with a 440 nm excitation filter and a 665 nm emission filter, calibrating the readings with pure chlorophyll *a* standards extracted from spinach (Sigma Chemical)

### Nutrient Limitation Experiments

I assessed nutrient limitation by carrying out small-scale nutrient addition experiments [54], [55] and measuring short-term algal accrual rates in two years (2001, 2002). I filled 35 ml black 35 mm film canisters with agar (20 g/l). A 2.6 mm diameter porous glass disc (crucible cover, Leco) was then placed on top of the agar to serve as an algal growth substrate. A 2.5 cm hole was punched in the lid of the canister to allow water exchange and the lid was snapped onto the top of the canister so that the disc fit snugly in the cap. The agar in each canister contained one of three compositions: agar only (controls), 0.5 M NaNO_3_ (+N treatment), or 0.05 M NaH_2_PO_4_ (+P treatment). The experimental canisters were then buried with the tops flush with the riverbed in a triangular pattern (controls at the upstream apex), and incubated for 2 weeks. They were then collected and chlorophyll *a* was extracted from the disc by placing it in 10 ml ethanol held within a closed, intact film canister for 24 hours at −10°C. Chlorophyll *a* concentration was then assessed fluorometrically as described above. Consistently higher chlorophyll *a* concentrations in +N or +P treatments relative to controls indicates nutrient limitation. A second experiment run in 2004 tested for N and P co-limitation using the same general experimental procedures. In this experiment, however, control canisters were filled with small dried pebbles from the riverbank, whereas nutrient addition canisters were filled with Osmocote® slow release fertilizer pellets (Scotts Company) with an 18∶6 N∶P ratio.

### Biotic Variables

To estimate algal standing crop, in each year of the study I placed 7.5×7.5 cm ceramic floor tiles on the river bottom to simulate natural cobbles [8], [56], [57], and incubated them in the river for 2 months during the summer. In late August, the tiles were collected and the attached algae were scraped using a combination of a razor blade and a toothbrush. Upon arrival in the laboratory, samples were diluted to 100 ml, homogenized with a hand blender, and split into two fractions. A 1 ml fraction was taken to measure chlorophyll *a* content, following the procedures outlined above. A second portion of the sample was filtered through a funnel containing glass fiber filter paper (Whatman GF/C), dried for 24 hours at 70°C, weighed, combusted at 500°C for 4 hours, and weighed again to derive algal ash free dry weight (AFDW).

To estimate aquatic invertebrate densities, two types of samples were taken in each year of the study. First, two 15×15 cm ceramic floor tiles were placed in the river at each site to simulate natural cobbles of standard size [8], [57]. After 2 months, the tiles were collected with a fine-mesh dip net and placed in a water-filled bucket. All invertebrates were dislodged from the tile by hand into the water, and the sample was filtered through 0.05 mm^2^ mesh. Samples were then preserved in 70% ethanol, enumerated and identified, generally to order or family, under a dissecting microscope in the laboratory. Second, a 10 (w)×15 (l)×10 (h) aluminum test tube basket was filled with small rocks (∼3 cm diameter) and buried in the sediment to mimic coarse gravel substrate. After 2 months, the basket was collected in a fine-mesh dip net, its contents dumped into a bucket with water, and the invertebrates on the rocks were dislodged with a swirling water motion. The water was then filtered through a 0.05 mm^2^ mesh filter, and the process was repeated four more times, increasing the degree of water agitation of each subsequent iteration. The sample was then processed like the invertebrate samples from the tiles. The average density of the tile and basket samples was used as the overall estimate of invertebrate abundance. This method of sampling was used in preference to alternatives such as Surber samplers because the base flow current speed at the sites at the time of sampling was generally too low (<10 cm/sec) to effectively use these methods, which require dislodged benthic invertebrates to be washed by the current into a downstream net.

To estimate fish density, I used a 60×60 cm quadrat constructed from PVC pipe and placed on the river bottom in cobble-dominated habitat [33]. After a 5-minute period that permitted fish to return to their normal behavior, observers standing on the shore counted the number of fish in the quadrat at the start of the observation plus the number of fish entering the quadrat over a 5-minute period. A set of four quadrats was taken at each site and the values from each quadrat were averaged to obtain a total fish density index. Because of the small size of the river in the study areas and the clarity of the water, fish throughout the width of the river were readily observed from shore, and quadrats sampled fish densities both in the middle and along the sides of the river. I calibrated the quadrat-based fish density index using an underwater video system by comparing the instantaneous number of fish recorded in a 0.2 m^2^ field of view at 1-minute intervals over a 20-minute period to quadrat counts taken in the areas at the same time. This procedure yielded the following calibration equation (r^2^ = 0.936, n = 10, P<0.0001):
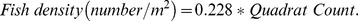



These passive censusing methods were developed and used in preference to other methods [58] because the river is too shallow and observer disturbance is too great to use snorkeling surveys, and invasive methods such as seining, electroshocking or rotenone application, which can all stress, damage or kill fish, are problematic when studying species of conservation concern.

At the same time that fish densities were being assessed, species composition was estimated independently by an observer on the shore using local keys [59], [60] as guides. Although coho salmon (*O. kitsuch*) are readily distinguished from the two trout species that live in the river because they have distinctive anal and dorsal fins and generally bar-shaped parr marks, free-swimming cutthroat trout (*O. clarkii*) and steelhead (*O. mykiss*) juveniles are more challenging to separate, in part because hybridization between the two species can occur [61]. Based on key characteristics and inspections of fish in the hand captured after I observed them in the water, trout with a lighter background coloration than coho salmon and extensive prominent dorsal spotting that tended to hold position in the channel were considered steelhead, whereas trout with darker background coloration than coho salmon and few (<5) large dorsal spots that tended to move more frantically than other fish between areas with cover were considered cutthroat trout. Because of the uncertainty in field identification, my estimates of the relative proportion of steelhead to cutthroat trout are subject to error and results focus on the two species lumped together as “trout”.

### Stable Isotope Analysis

Energy sources were traced from leaf fall, algal production and carcasses of adult salmon [62] to juvenile salmonids using N and C stable isotope methods [63], [64]. Algal samples for stable isotope analysis were collected by scraping a subsample of algae from a 7.5×7.5 cm area of tiles in the grazer exclusion buckets at each site. Hence, the algal community reflected both differences in light regime between experimental and control reaches, and also represented the algae potentially consumed by grazers, rather than the algae not consumed by grazers that remains outside the grazer exclusions. Leaves were collected for analysis at each site in leaf traps as described previously and were analyzed as a multi-species group in the proportions caught in the leaf traps. Five juvenile coho salmon (*Oncorhynchus kisutch*) were collected at each site, measured (standard length), and dissected to separate muscle from gut tissue. For adult coho salmon, muscle tissue of fish captured by hook and line in the Strait of Juan de Fuca were used. Samples of all organisms were dried at 40°C in the laboratory, ground into a fine powder using a Wiley mill, and shipped to the University of Utah Stable Isotope Ratio Facility for Environmental Research, where they were combusted and analyzed with a continuous flow mass spectrometer.

### Statistical Analysis

I analyzed most of the data using paired t-tests, pairing the closest control and experimental reaches to control for possible effects of downstream location and the presence of experimental manipulations upstream. In all cases, I analyzed the mean response at each site averaged across all years sampled to avoid temporal pseudoreplication. When variance among treatments was unequal, data were log transformed before applying paired t-tests. To maximize statistical power, in cases where there were clear a priori directional predications (i.e., PAR, UV, canopy cover, leaf input), one-tailed tests were used. For nutrient limitation experiments, a split-plot ANOVA design was used (nutrient manipulations nested within riparian treatments; [33]).

Stable isotope data were analyzed using the Bayesian algorithm MixSIR [65], which accounts for measured variability in isotopic signatures and uncertainty in isotopic enrichment with each trophic transfer. Analyses were performed on each treatment, using treatment-specific data for juvenile salmon and algae. To estimate enrichment for each trophic transfer, I used the mean and standard deviation of trophic enrichment values from 56 studies for N and 85 studies for C, summarized in [63]. I assumed two trophic transfers from energy sources to juvenile salmon, as juvenile salmon are not known to be herbivorous. Alternative analyses assuming a single trophic transfer from salmon carcasses to juvenile salmon yielded qualitatively similar results. In all analyses, I assumed a uniform prior distribution. For each analysis, 1 million randomly chosen diet combinations were drawn to estimate likelihoods of different parameters given the data and estimate posterior probabilities of different energy source contributions.

## References

[1] Berg DR (1995) Riparian silvicultural system design and assessment in the Pacific Northwest Cascade Mountains, USA. Ecol Appl 5: 87–96.

[2] Kareiva P, Marvier M (2000) Recovery and management options for spring/summer chinook salmon in the Columbia River basin. Science 290: 977–979. 11062128 10.1126/science.290.5493.977

[3] Walters CJ, Christensen V, Martell SJ, Kitchell JF (2005) Possible ecosystem impacts of applying MSY policies from single-species assessments. ICES J Mar Sci 62: 558–568.

[4] Crowder LB, Hazen EL, Avissar N, Pjorkland R, Latanich K, et al (2008) The impacts of fisheries on marine ecosystems and the transition to ecosystem-based management. Ann Rev Ecol Evol Syst 39: 259–278.

[5] Bothwell ML, Sherbot DMJ, Pollock CM (1994) Ecosystem response to solar ultraviolet-B radiation: Influence of trophic-level interactions. Science 265: 97–100. 17774696 10.1126/science.265.5168.97

[6] Jackson JBC, Kriby MX, Berger WH, Bjorndal KA, Botsford LW, et al (2001) Historical overfishing and the recent collapse of coastal ecosystems. Science 293: 629–638. 11474098 10.1126/science.1059199

[7] Mangel M, Levin P (2005) Regime, phase and paradigm shifts: Making community ecology the basic science for fisheries. Phil Trans Royal Soc London, Series B 360: 95–105. 10.1098/rstb.2004.1571 PMC1636097 15713590

[8] Wootton JT, Parker MS, Power ME (1996) Effects of disturbance on river food webs. Science 273: 1558–1561.

[9] Levin PS, Fogarty MJ, Murawski SA, Fluharty D (2009) Integrated Ecosystem Assessments: Developing the Scientific Basis for Ecosystem-Based Management of the Ocean. PLoS Biol 7 1: e1000014 doi:10.1371/journal.pbio.1000014. 10.1371/journal.pbio.1000014 PMC2628402 19166267

[10] Moring JR, Lantz RL (1975) The Alsea watershed study: Effects of logging on the aquatic resources of three headwater streams of the Alsea River, Oregon. Part I – biological studies. Fishery Research Report No. 9. Corvallis, OR: Oregon Department of Fish and Wildlife.

[11] Hartman GF, Scrivener JC (1990) Impacts of forestry practices on a coastal stream ecosystem, Carnation Creek, British Columbia. Can J Fish Aquat Sci 223: 1–148.

[12] Holtby LB (1988) Effects of logging on stream temperatures in Carnation Creek, British Columbia, and associated impacts on the coho salmon (*Oncorhynchus kisutch*). Can J Fish Aquat Sci 45: 502–515.

[13] Kiffney PM, Richardson JS, Bull JP (2003) Responses of periphyton and insects to experimental manipulation of riparian buffer width along forest streams. J Appl Ecol 40: 1060–1076.

[14] Sedell JR, Reeves GH, Hauer FR, Stanford JA, Hawkins CP (1990) Role of refugia in recovery from disturbances: modern fragmented and disconnected river systems. Environ Manage 14: 711–724.

[15] Fausch KD, Northcote TG (1992) Large woody debris and salmonid habitat in a small coastal British Columbia stream. Can J Fish Aquat Sci 49: 682–693.

[16] Ralf SC, Poole GC, Conquest LL, Naiman RJ (1994) Stream morphology and woody debris in logged and unlogged basins of western Washington. Can J Fish Aquat Sci 51: 37–51.

[17] Montgomery DR, Abbe TB, Buffington JM, Peterson NP, Schmidt KM, et al (1996) Distribution of bedrock and alluvial channels in forested mountain drainage basins. Nature 381: 587–588.

[18] Roni P, Beechie TJ, Bilby RE, Leonetti FE, Pollock MM, et al (2002) A review of stream restoration techniques and a hierarchical strategy for prioritizing restoration in Pacific Northwest watersheds. N Am J Fish Manage 22: 1–20.

[19] Likens GE, Bormann FH, Johnson NM, Fisher DW, Pierce RS (1970) Effects of forest cutting and herbicide treatment on nutrient budgets in the Hubbard Brook watershed-ecosystem. Ecol Monogr 40: 23–47.

[20] Murphy ML, Hawkins CP, Anderson NH (1981) Effects of canopy modification and accumulated sediment on communities. Trans Am Fisheries Soc 110: 469–478.

[21] Bilby RE, Bisson PA (1992) Allochthonous versus autochthonous organic matter contributions to the trophic support of fish populations in clear-cut and old-growth forested streams. Can J Fish Aquat Sci 49: 540–551.

[22] Wootton JT, Power ME (1993) Productivity, consumers, and the structure of a river food chain. Proc Natl Acad Sci USA 90: 1384–1387. 11607368 10.1073/pnas.90.4.1384 PMC45877

[23] Yeakley JA, Meyer JL, Swank WT (1994) Hillslope nutrient flux during near-stream vegetation removal: I. A multi-scaled modeling design. Water Air Soil Poll 77: 229–246.

[24] Wallace JB, Eggert SL, Meyer JL, Webster JR (1997) Multiple trophic levels of a forest stream linked to terrestrial litter inputs. Science 277: 102–104.

[25] Kiffney PM, Clements WH, Cady TA (1997) Influence of ultraviolet radiation on the colonization dynamics of a Rocky Mountain stream benthic community. J N Am Benthol Soc 16: 520–530.

[26] Wigington PJ, Church MR, Strickland TC, Eshleman KN, Van Sickle J (1998) Autumn chemistry of Oregon Coast Range streams. J Am Water Res Assoc 34: 1035–1049.

[27] Wilzbach MA, Harvey BC, White JL, Nakamoto RJ (2005) Effects of riparian canopy opening and salmon carcass addition on abundance and growth of resident salmon. Can J Fish Aquat Sci 62: 58–67.

[28] Sedell JR, Froggatt JL (1984) Importance of streamside forests to large rivers: the isolation of the Willamette River, Oregon, USA, from its floodplain by snagging and streamside forest removal. Verh Internat Verein Limnol 22: 1828–1834.

[29] Gregory SV, Swanson FJ, McKee WA, Cummins KW (1991) An ecosystem perspective of riparian zones. Bio Science 41: 540–551.

[30] Perrin CJ, Bothwell ML, Slaney PA (1987) Experimental enrichment of a coastal stream in British Columbia: effects of organic and inorganic additions on autotrophic periphyton production. Can J Fish Aquat Sci 44: 1247–1256.

[31] Johnston NT, Perrin CJ, Slaney PA, Ward BR (1990) Increased juvenile salmonid growth by whole-river fertilization. Can J Fish Aquat Sci 47: 862–872.

[32] Slaney PA, Rublee BO, Perrin CJ, Goldberg H (1994) Debris structure placements and whole-river fertilization for salmonids in a large regulated stream in British Columbia. Bull Mar Sci 55: 1160–1180.

[33] Wootton JT (2012) Effects of timber harvest on river food webs: physical, chemical and biological responses. PLoS ONE 7 9: e43561 doi:10.1371/journal.pone.0043561. 22957030 10.1371/journal.pone.0043561 PMC3434149

[34] Harpole WS, Ngai JT, Cleland EE, Borer ET, Bracken MES, et al (2011) Nutrient co-limitation of primary producer communities. Ecol Lett 14: 852–862. 21749598 10.1111/j.1461-0248.2011.01651.x

[35] MacLoed NA, Barton DR (1998) Effects of light intensity, water velocity, and species composition on carbon and nitrogen stable isotope ratios in periphyton. Can J Fish Aquat Sci 55: 1919–1925.

[36] Findlay JC, Power ME, Cabaña G (1999) Effects of water velocity on algal carbon isotope ratios: Implications for river food web studies. Limnol Oceanogr 44: 1198–1203.

[37] Janisch JE, Wondzell SM, Ehlinger WJ (2012) Headwater stream temperature: Interpreting response after logging, with and without riparian buffers, Washington, USA. Forest Ecol Manag 270: 302–313 DOI: 10.1016/j.foreco.2011.12.035.

[38] Finlay JC, Hood JM, Limm MP, Power ME, Schade JD, et al (2011) Light-mediated thresholds in stream-water nutrient composition in a river network. Ecology 92: 140–150 DOI:10.1890/09-2243.1. 21560684 10.1890/09-2243.1

[39] Heifetz J, Murphy ML, Koski KV (1986) Effects of logging on winter habitat of juvenile salmonids in Alaskan USA streams. N Am J Fisheries Management 6: 52–58.

[40] Esseen PA (1994) Tree mortality patterns after experimental fragmentation of an old-growth conifer forest. Biol Cons 68: 19–28.

[41] Roni P, Quinn TP (2001) Density and size of juvenile salmonids in response to placement of large woody debris in western Oregon and Washington streams. Can J Fish Aquat Sci 58: 282–292.

[42] Hartman GF, Brown TG (1987) Use of small, temporary, floodplain tributaries by juvenile salmonids in a West Coast rain-forest drainage basin, Carnation Creek, British Columbia. Can J Fish Aquat Sci 44: 262–270.

[43] Lin BH, Williams NA (1988) Specifying a functional form for the influence of hatchery smolt release on adult salmon production. Fisheries Bull 86: 655–662.

[44] Emlen JM, Reisenbichler RR, McGie AM, Nickelson TE (1990) Density-dependence at sea for coho salmon *Oncorhynchus kisutch* . Can J Fish Aquat Sci 47: 1765–1772.

[45] Nickelson TE, Lawson PW (1998) Population viability of coho salmon, *Oncorhynchus kisutch*, in Oregon coastal basins: Application of a habitat-based life cycle model. Can J Fish Aquat Sci 55: 2383–2392.

[46] McHenry ML, Shott E, Conrad RH, Grette GB (1998) Changes in the quantity and characteristics of large woody debris in streams of the Olympic Peninsula, Washington, USA (1982–1993). Can J Fish Aquat Sci 55: 1395–1407.

[47] Hyatt TL, Naiman RJ (2001) The residence time of large woody debris in the Queets River, Washington, USA. Ecol Appl 11: 191–202.

[48] Webster JR, Benfield EF (1986) Vascular plant breakdown in freshwater ecosystems. Ann Rev Ecol Syst 17: 567–594.

[49] Rosemond AD, Mulholland PJ, Brawley SH (2000) Seasonally shifting limitation of stream periphyton: Response of algal populations and assemblage biomass and productivity to variation in light, nutrients, and herbivores. Can J Fish Aquat Sci 57: 66–75.

[50] Nakano S, Murakami M (2001) Reciprocal subsidies: Dynamic interdependence between terrestrial and aquatic food webs. Proc Natl Acad Sci U S A 98: 166–170. 11136253 10.1073/pnas.98.1.166 PMC14562

[51] Rosenfeld JS, Hudson JJ (1997) Primary production, bacterial production, and invertebrate biomass in pools and riffles in southern Ontario streams. Arch Hydrobiol 139: 301–316.

[52] Whitledge GW, Rabeni CF (2000) Benthic community metabolism in three habitats in an Ozark stream. Hydrobiologia 437: 165–170.

[53] UNESCO (1994) Protocols for the Joint Global Ocean Flux Study (JGOFS) core measurements. Paris: IOC Manual and Guides Number 29.

[54] Marks JC, Power ME, Parker MS (2000) Flood disturbance, algal productivity, and interannual variation in food chain length. Oikos 90: 20–27.

[55] Gibeau GG Jr, Miller MC (1989) A micro-bioassay for epilithon using nutrient-diffusing artificial substrata. J Freshwater Ecol 5: 171–176.

[56] Tuchman ML, Stevenson RJ (1980) Comparison of clay tile, sterilized rock, and natural substrate diatom communities in a small stream in southeastern Michigan, USA. Hydrobiologia 75: 73–79.

[57] Lamberti GA, Resh VH (1985) Comparability of introduced tiles and natural substrates for sampling lotic bacteria algae and macroinvertebrates. Freshwater Biol 15: 21–30.

[58] Li HW, Li JL (1996) Fish community composition. In: Hauer FR, Lamberti GA, editors. Methods in stream ecology. New York: Academic Press. Pp 391–406.

[59] Wydoski RS, Whitney RR (1979) Inland fishes of Washington. Seattle: University of Washington Press.

[60] Pollard WR, Hartman GF, Groot C, Edgell P (1997) Field identification of coastal juvenile salmonids. Madeira Park, BC: Harbour Publishing.

[61] Baumsteiger J, Hankin D, Loudenslager EJ (2005) Genetic Analyses of Juvenile Steelhead, Coastal Cutthroat Trout, and Their Hybrids Differ Substantially from Field Identifications. Trans Am Fisheries Soc 134: 829–840.

[62] Bilby RE, Fransen BR, Bisson PA, Walter JK (1998) Response of juvenile coho salmon (*Oncorhynchus kisutch*) and steelhead (*Onchorhynchus mykiss*) to the addition of salmon carcasses to two streams in southwestern Washington. Can J Fish Aquat Sci 55: 1909–1918.

[63] Peterson BJ, Fry B (1987) Stable isotopes in ecosystem studies. Ann Rev Ecol Syst 18: 293–320.

[64] Lajtha K, Michener RH (1994) Stable isotopes in ecology and environmental science. London: Blackwell.

[65] Moore JW, Semmens BX (2008) Incorporating uncertainty and prior information into stable isotope mixing models. Ecol Lett 11: 470–480. 18294213 10.1111/j.1461-0248.2008.01163.x

